# Unraveling the SARS-CoV-2 spike protein long-term effect on neuro-PASC

**DOI:** 10.3389/fncel.2024.1481963

**Published:** 2024-12-18

**Authors:** Filipe Menezes, Julys da Fonseca Palmeira, Juliana dos Santos Oliveira, Gustavo Adolfo Argañaraz, Carlos Roberto Jorge Soares, Otávio Toledo Nóbrega, Bergmann Morais Ribeiro, Enrique Roberto Argañaraz

**Affiliations:** ^1^Biotechnology Center, Instituto de Pesquisas Energéticas e Nucleares, IPEN-CNEN/SP, São Paulo, Brazil; ^2^Laboratory of Molecular Neurovirology, Faculty of Health Science, University of Brasília, Brasília, Brazil; ^3^Programa de Pós-graduação em Ciências Médicas da Faculdade de Medicina, University of Brasília, Brasília, Brazil; ^4^Baculovirus Laboratory, Department of Cell Biology, University of Brasília, Brasília, Brazil

**Keywords:** neuro-PASC, pathophysiology, SARS-CoV-2 spike protein, blood–brain barrier, SARS-CoV-2 receptors

## Abstract

The persistence or emergence of long-term symptoms following resolution of primary SARS-CoV-2 infection is referred to as long COVID or post-acute sequelae of COVID-19 (PASC). PASC predominantly affects the cardiovascular, neurological, respiratory, gastrointestinal, reproductive, and immune systems. Among these, the central nervous system (CNS) is significantly impacted, leading to a spectrum of symptoms, including fatigue, headaches, brain fog, cognitive impairment, anosmia, hypogeusia, neuropsychiatric symptoms, and peripheral neuropathy (neuro-PASC). However, the risk factors and pathogenic mechanisms responsible for neuro-PASC remain unclear. This review hypothesis discusses the leading hypotheses regarding the pathophysiological mechanisms involved in long COVID/PASC, focusing on neuro-PASC. We propose vascular dysfunction mediated by activation of astrocytes and pericytes followed by blood–brain barrier (BBB) disruption as underlying pathophysiological mechanisms of neurological manifestations. Additionally, we provide insights into the role of spike protein at the blood–brain interface. Finally, we explore the potential pathogenic mechanisms initiated by the interaction between the spike protein and cellular receptors at the brain endothelial and tissue levels.

## 1 Introduction

Severe acute respiratory syndrome coronavirus 2 (SARS-CoV-2), responsible for coronavirus disease 2019 (COVID-19), has resulted in over 0.7 billion infections and almost 7 million deaths (WHO, 2023). The rapid spread of the virus before symptom onset, combined with acute clinical complications such as severe inflammation and disseminated intravascular coagulation, along with the accelerated emergence of variants of concern (VOCs) associated with intra-host viral evolution, have been primary contributors to the high morbidity and mortality rates (Chaguza et al., 2022). Despite the success of vaccination efforts in reducing mortality and transmission rates in the last 3 years, 10–40% of convalescent patients have experienced long COVID syndrome or post-acute sequelae of SARS-CoV-2 infection (PASC) (Zheng et al., 2022). Additionally, a significant subset of long-term COVID patients, up to 40%, have developed neurological and psychiatric symptoms (Taquet et al., 2021). The emergence of long COVID/PASC, together with VOCs and the increasing number of global infections, highlights the urgent need to understand the mechanisms underlying long COVID/PASC. In this context, this review hypothesis focuses on exploring the underlying mechanisms of neuro-PASC, primarily related to the SARS-CoV-2 spike protein (S).

## 2 Post-acute COVID-19 sequelae (PASC): a new disease?

The COVID-19 pandemic has evolved into a significant public health issue, manifesting as long COVID or PASC. The WHO defines PASC or long COVID as a new clinical entity characterized by the persistence of symptoms for at least 3 months after the initial viral infection, lasting at least 2 months, and not explained by an alternative diagnosis (Al-Aly et al., 2021; WHO, 2021; Soriano et al., 2022). It is estimated that long COVID/PASC affects at least 10% of all infected patients, equating to approximately 65 million individuals worldwide, with cases increasing daily (Tran et al., 2022; Davis et al., 2023). Long-COVID/PASC incidence ranges from 50 and 70% in hospitalized and not hospitalized patients, respectively, for 2 years after acute infection (Fernandez-de-Las-Penas et al., 2022b; Bull-Otterson et al., 2022), and around 10–12% in vaccinated patients (Ayoubkhani et al., 2022). Symptoms can persist for up to 2 or 3 years and may vary over time (Sudre et al., 2021; Davis et al., 2023). Recent findings suggest that the duration of long COVID is extending. In a study involving 135,161 infected patients followed for 3 years, a decrease in the number of infected patients, mortality risk, and PASC was observed over time (Cai et al., 2024). While the risk of death diminishes after 1 year in non-hospitalized patients, it persists beyond 3 years in hospitalized patients, along with an increased risk of developing PASC.

PASC is characterized by a variety of long-lasting symptoms, with the most common being shortness of breath, headache, fatigue, cognitive dysfunction (memory impairment and lack of concentration), anxiety, myalgia, joint pain, smell and taste dysfunction, cough, insomnia, rhinorrhea (Lopez-Leon et al., 2021). Furthermore, neurological and neurodegenerative manifestations have been reported in patients without previous clinical history including encephalopathy, stroke, seizures, encephalitis, and Guillain-Barré syndrome. It is important to highlight that more than 50 symptoms have been documented (Soriano et al., 2022). Importantly, the types and severity of COVID-19 symptoms can depend on the SARS-CoV-2 variant of infection (Bouzid et al., 2022; Whitaker et al., 2022). Thus, the great variability in symptoms and severity, as well as duration, led to classifying PASC into different types (Fernandez-de-Las-Penas et al., 2021). However, the Wuhan variant has been shown to induce a greater number of symptoms when compared to the Alpha or Delta variants (Fernandez-de-Las-Penas et al., 2022a).

### 2.1 Neuro-PASC

Neurological manifestations are among the primary occurrences of long COVID (Ding and Zhao, 2023). A study involving 226 patients who survived COVID-19 pneumonia evaluated them between 1 and 3 months after discharge (Mazza et al., 2021). It found that 78% of patients had disabilities in at least one cognitive domain, 50–57% had impaired psychomotor coordination, and 20–70% exhibited cognitive deficits during the COVID-19 acute phase. In a recent cross-sectional study of 57 hospitalized patients, over 80% exhibited significant cognitive impairment, particularly in attention and executive function (Jaywant et al., 2021). Another study followed 18 patients with mild to moderate COVID-19 and found similar issues (Ritchie and Chan, 2021). A different study following 13,001 individuals found significantly higher rates of memory problems 8 months after infection (Garrigues et al., 2020). Then in a cohort study, symptoms such as anosmia, ageusia, memory loss, and headache persisted beyond 60 days, with memory deficits continuing up to the third month after infection (Han et al., 2022). Proposed mechanisms contributing to long-term cognitive impairment include blood–brain barrier (BBB) vascular disruption, neuroinflammation, synaptic dysfunction, disturbed neurotransmitter release, and neuronal loss (Shabani et al., 2023).

## 3 Understanding the pathophysiology of neuro-PASC

Although PASC is a multiorgan disease, post-acute neurological sequelae are prevalent and significantly impact the quality of life, making the CNS an important target for investigation (Moghimi et al., 2021). The pathogenic mechanisms responsible for PASC, particularly neuro-PASC, remain largely unknown. Key questions include whether certain mechanisms are responsible for certain symptoms and whether multiple mechanisms might act in concert to produce PASC. The primary underlying mechanisms of PASC may include both direct effects due to viral brain infection, or persistence of viral components (Liotti et al., 2021; Chen et al., 2023), as well as indirect effects stemming from secondary mechanisms. These secondary mechanisms may include: (i) impairment of the cerebral vasculature (VanElzakker et al., 2024); (ii) Immune dysregulation, which can lead to inflammation, hypoxia, complement activation, thromboinflammation, autoimmunity, re-activation of neurotrophic viruses, and hormonal dysregulation (Pretorius et al., 2021; Adingupu et al., 2023). The widespread expression of the main SARS-CoV-2 receptor, the angiotensin-converting enzyme 2 (ACE2) molecule, in both neural and endothelial cells (EC), renders the CNS susceptible to SARS-CoV-2 infection (Baig et al., 2020). Although several autopsy-based studies have described neurological alterations in various brain regions, evidence of viral replication or presence in the brain or cerebrospinal fluid (CSF) is infrequent (Pattanaik et al., 2023). These findings raise questions about whether the limited viral detection is due to the low sensitivity of detection methods or the sampling timing, and whether the presence of detected viral RNA indicates persistent viral replication. In this context, mechanisms independent of infection and viral replication may play a leading role in neuro-PASC effects (Hellmuth et al., 2021). Moreover, the persistence of SARS-CoV-2/S protein in the brain and its interaction with cellular receptors at the endothelial and tissue levels may have a relevant role in neuro-PASC (Montezano et al., 2023).

### 3.1 Neuro-PASC: a disease at the blood–brain interface?

The regions at the interface between the blood and the brain are primarily composed of the BBB, choroid plexus, and circumventricular organs (CVOs) (Miyata, 2022). Given its unique structure, the BBB exhibits highly selective permeability, limiting the passage of substances and pathogens such as SARS-CoV-2 from the blood to the brain parenchyma, while also playing a fundamental role in CNS homeostasis (Wu et al., 2023). It is proposed that BBB dysfunction not only results from, but also causally contributes to the pathogenesis of neurological disorders like Alzheimer’s disease, Parkinson’s disease, multiple sclerosis, and neuro-PASC (Xiao et al., 2020; Finsterer et al., 2022). Spike-mediated damage to vessels forming the BBB and enhanced immune responses have been identified as major causes of chronic neurological symptoms in PASC (Suprewicz et al., 2023).

Cognitive impairment is one of the most prevalent symptoms of PASC. The dysfunction of BBB can lead to cerebral hypoperfusion, hypometabolism, and cognitive impairment in individuals with PASC. Several recent studies using advanced neuroimaging and processing techniques have reported hypometabolism in various brain regions in patients with PASC experiencing persistent functional symptoms, including cognitive deficits (Hosp et al., 2021). It has recently been demonstrated that BBB disruption and sustained systemic inflammation are evident in patients with cognitive impairment positive for PASC, commonly referred to as “brain fog” (Alquisiras-Burgos et al., 2021; Greene et al., 2024). Using a longitudinal design and investigating mechanistic pathways associated with the development of cognitive impairment in PASC individuals, an association with white matter integrity loss has been suggested, potentially mediated by BBB compromise and associated glutamatergic excitotoxicity (Chaganti et al., 2024). CVOs, neural structures around the third and fourth ventricles, harbor vessels similar to the choroid plexus, lacking a BBB. This allows them to sense stimulatory molecules in the bloodstream but also increases their susceptibility to pathogen exposure (Jeong et al., 2021). Nevertheless, attacks on CVOs by pathogens are rarely described. For instance, trypanosomes present in the choroid plexus can enter the ventricles and initiate accelerated infiltration of T cells and parasites in periventricular areas, eventually entering the brain parenchyma from the median eminence (a CVO located at the base of the third ventricle) into the boundary to the hypothalamic arcuate nuclei protected by the BBB. This process provides a pathway for pathogens to infiltrate brain regions connected to circadian rhythm and sleep–wake regulation networks, to which other CVOs are also connected (Bentivoglio et al., 2018). Significant cellular disruptions in COVID-19 patients indicate that choroid plexus barrier cells detect and transmit peripheral inflammation to the brain, with peripheral T cells infiltrating the parenchyma (Yang et al., 2021).

A study using a pseudovirus containing SARS-CoV-2 S protein demonstrated that the spike protein damages the choroid plexus epithelium, leading to leakage through this critical barrier, which normally prevents the entry of pathogens, immune cells, and cytokines into the CSF and the brain (Pellegrini et al., 2020). From the CSF which bathes the ventricles and meninges, the S protein can accumulate along the skull-meninges-brain axis and potentially have implications for long-term neurological complications, associated with pathways related to neutrophils and dysregulation of proteins involved in phosphatidylinositol 3-kinase (PI3K)-AKT signaling, complement activation, and coagulation. Additionally, the S protein may travel from the edges of the CNS to the brain parenchyma and directly affect brain tissue. A recent study conducted by Rong and colleagues showed that the Spike protein remains in the skull-meninges-brain axis in patients with COVID-19 long after the virus has been cleared. Elevated levels of neurodegeneration biomarkers were also detected in the cerebrospinal fluid (CSF) of patients with Long COVID. In an animal model, the Spike protein, specifically the S1 subunit, was shown to alter the proteome in the skull-meninges-brain axis and induce anxiety-like behaviors. The study identified differential expression of proteins associated with the interleukin (IL)-18 signaling pathway, as well as changes in proteins related to the MAPK and (PI3K)-AKT signaling pathways, and proteins involved in neutrophil extracellular trap (NET) formation. Additionally, the Spike S1 subunit led to an increase in caspase-3 activity. The authors proposed that the Spike protein could travel from the cranial spinal cord to the meninges and brain parenchyma, thereby contributing to long-term complications (Rong et al., 2024).

SARS-CoV-2 viral particles transmitted through the blood can also enter the brain by extravasating through fenestrated vessels of CVOs, thereby bypassing the BBB. This hypothesis is supported by the abundance of viral markers in the median eminence of the hypothalamus from post-mortem COVID-19 patients, including edema and neuronal degeneration (Pal, 2020; Sauve et al., 2023). Additionally, immuno-histochemical studies detected high ACE2 expression in CVOs closely connected to the hypothalamus and the hypothalamus (Ong et al., 2022).

The median eminence is composed of specialized ependymoglial cells called tanycytes. The spike protein was observed at extremely high levels in the terminal feet of tanycytes in a patient who died of severe COVID-19, suggesting internalization by the terminal feet of tanycytes at the level of fenestrated capillaries, but also subsequent transfer to other types of cells (Ternier et al., 2020). Permeation of circulating S protein through the median eminence to the hypothalamic nuclei could promote neuronal degeneration in this region and impair hormonal regulation by the central nervous system (Ong et al., 2022).

#### 3.1.1 Spike protein and blood–brain interface dysfunction hypothesis

**Figure 1:**
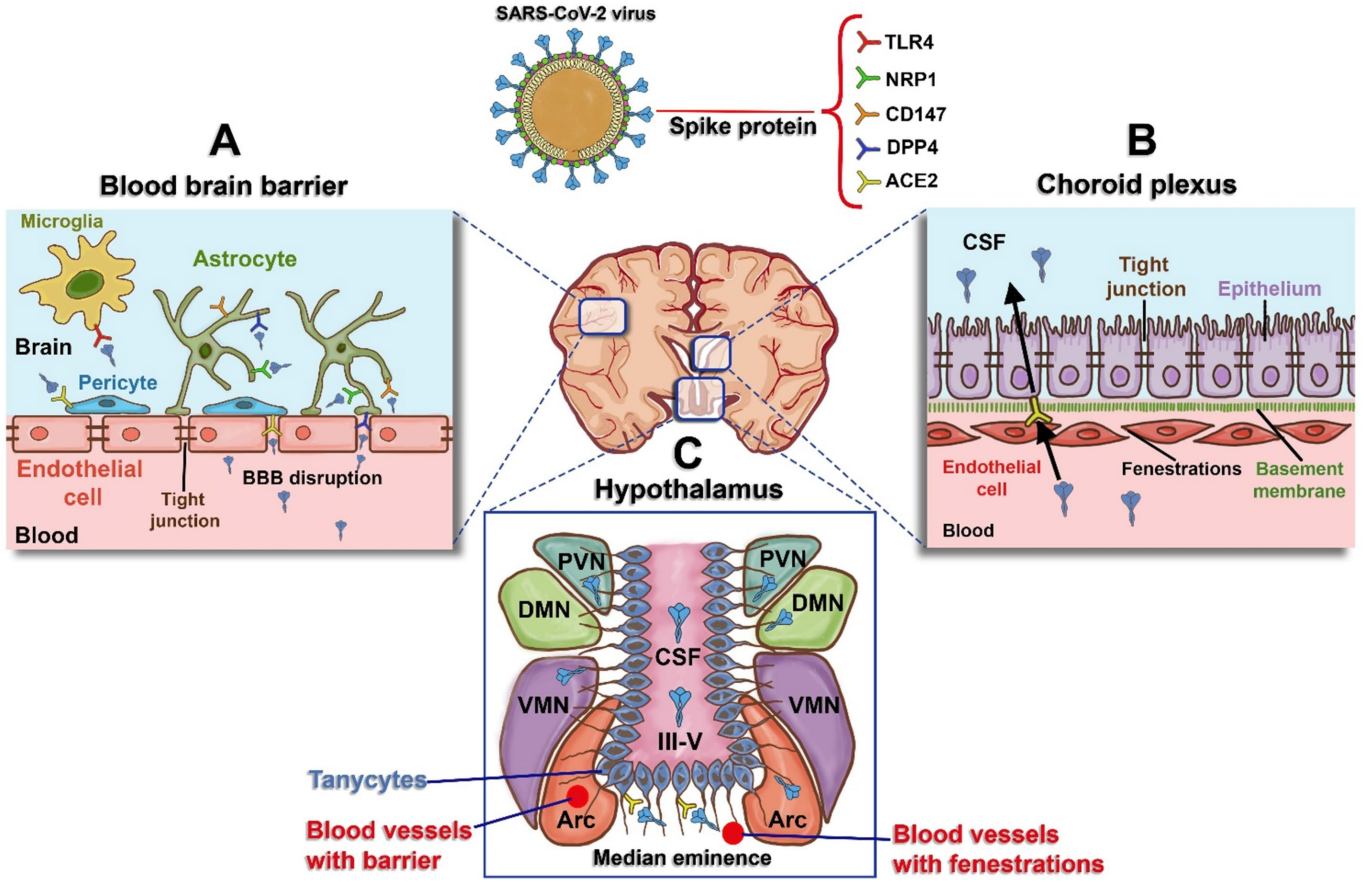
Schematic representation of blood and the brain regions interface and receptors expressed for the spike protein. **(A)** Blood–Brain Barrier (BBB): The BBB is formed by tight junctions in endothelial cells. The spike protein induces damage to these tight junctions, resulting in BBB disruption, increased permeability, and access to receptors present in astrocytes (human dipeptidyl peptidase - DPP4, Neuropilin-1 - NRP1, and Cluster of Differentiation - CD147), pericytes (Angiotensin-Converting Enzyme 2 - ACE2), and microglia (Toll-like Receptor 4 - TLR4). **(B)** Choroid Plexus: Specialized tissue located in the wall of the fourth ventricle, composed of endothelial cells that are more permeable than those of the BBB, with gaps known as fenestrations. This enables easier movement for the epithelium on the apical side, which expresses ACE2, and this acts as a potential entry route for spike protein into the cerebrospinal fluid (CSF). **(C)** Hypothalamus: Hypothalamic tanycytes can be observed around the third ventricle (III-V), expressing ACE2 and transferring spike protein to the regions of the arcuate nucleus (ARC), ventromedial nucleus (VMN), dorsomedial nucleus (DMN), and periventricular nucleus (PVN). Figure created with BioRender.com.

Some researchers suggest that SARS-CoV-2’s ability to infect the human brain through ACE2 expression, along with prolonged viral infection and the direct effects of the spike protein on neural tissue, could be linked to long-term neurological consequences (Datta et al., 2021; Ding and Zhao, 2023). However, the question of whether SARS-CoV-2 directly infects neurons remains debated (Solomon et al., 2020), and there is limited or no evidence of active viral replication in the brains of individuals with Post-Acute Sequelae of SARS-CoV-2 (PASC) (van den Bosch et al., 2022). In this context, we propose that the spike protein directly affects neural tissue and may, at least in part, contribute to the neuropathogenic mechanisms observed in NeuroPASC, especially in areas with high ACE2 expression. As discussed further below, viral receptors are more abundantly expressed in the cells forming the blood–brain interface compared to neurons themselves (Lukiw et al., 2022; Ong et al., 2022; Potokar et al., 2023). PASC-positive individuals often show persistent or elevated levels of circulating spike protein for up to a year or longer after acute infection (Craddock et al., 2023; Schultheiß et al., 2023; Swank et al., 2023). This suggests that the spike protein may be stored in cellular reservoirs, released into the bloodstream, and subsequently absorbed by other regions, potentially causing complications through endothelial damage (Cao et al., 2023). This mechanism could explain the BBB disruption and ongoing systemic inflammation observed in patients with cognitive dysfunction. In addition to the BBB, the blood-cerebrospinal fluid barrier (BCSFB), which includes structures like the choroid plexus and circumventricular organs (CVOs) such as the median eminence of the hypothalamus, may also suffer persistent damage from the spike protein (Pellegrini et al., 2020; Ong et al., 2022). Damage to these areas is closely associated with the symptoms of NeuroPASC. In summary, we hypothesize that the damage caused by circulating spike protein, combined with high permeability in certain tissues and elevated ACE2 expression, leads to sustained interactions with other viral receptors or coreceptors in cells of the blood–brain interface, such as pericytes and astrocytes of the BBB, epithelial cells of the choroid plexus, tanycytes, and hypothalamic neurons. These interactions could explain many of the primary symptoms observed in NeuroPASC (Figure 1).

### 3.2 Hypothalamic–pituitary axis dysfunction and NeuroPASC symptoms

The ACE2 protein is highly expressed in the paraventricular nucleus of the hypothalamus (PVH). The PVH is a stress-responsive center in the brain, controlling pre-ganglionic sympathetic neurons and serving as a source of corticotropin-releasing hormone, which induces adrenocorticotropic hormone (ACTH) secretion from the anterior pituitary (Alzahrani et al., 2021). The adrenocortical response was reported to be impaired in patients with acute COVID-19 infection, with a large percentage of patients having plasma cortisol and ACTH levels consistent with central adrenal insufficiency (Alzahrani et al., 2021). It is proposed that ACE2 function in CVOs in the PVH could be diminished by S protein binding, resulting in increased pre-sympathetic/neuroendocrine PVH activity and affecting hypothalamic–pituitary–adrenal axis activity. Therefore, the function of ACE2-positive neurons in the PVH may be affected by the SARS-CoV-2 spike protein, interfering with Ang II function modulation and reducing stress/anxiety modulation in COVID-19 patients (Ong et al., 2022).

Damage to the hypothalamus by COVID-19 can lead to dysfunction in hypothalamic–pituitary-testicular axis (Ardestani Zadeh and Arab, 2021), potentially affecting testicular function (Selvaraj et al., 2021). Additionally, infection of gonadotropin-releasing hormone (GnRH) neurons, which are crucial for regulating reproduction, or tanycytes, multifunctional hypothalamic glia that interact with GnRH neuron terminals, could contribute to hypogonadotropic hypogonadism (Sauve et al., 2023). A causal link has been demonstrated between GnRH loss and cognitive deficits during pathological aging, including Down syndrome and Alzheimer’s disease (Sunada et al., 2022). Olfactory and cognitive changes that persist in some COVID-19 patients, along with long-term hypogonadism in men infected with SARS-CoV-2, resemble the consequences of GnRH deficiency. This suggests that neuroinvasion of the GnRH system may underlie certain post-COVID symptoms, potentially leading to accelerated or exacerbated cognitive decline (Sauve et al., 2023). Furthermore, GnRH neurons were found to be dying in the post-mortem brains of COVID-19 patients, significantly reducing GnRH expression. Fetal human olfactory and vomeronasal epithelia, and fetal GnRH neurons (proceeding of these epithelia) also appeared to be susceptible to infection (Sauve et al., 2023).

Chronic Fatigue Syndrome (CFS) remains a central and common complaint among post-COVID patients (Nalbandian et al., 2023). Low-grade chronic neuroinflammation induced by the SARS-CoV-2 virus may explain chronic fatigue in individuals without chronic cardiac, pulmonary, or renal dysfunction (Mueller et al., 2020). Additionally, endocrine dysfunctions, such as hypercortisolism, hypothyroidism, or disruption of the HPA axis may also contribute to CFS (Bansal et al., 2022). A study of patients recovering from previous SARS-CoV infection, found that hypercortisolism persisted for up to 1 year in most patients, along with central hypothyroidism and low dehydroepiandrosterone sulfate (DHEAS) in some patients, supporting chronic deficiency of corticotropin (CRH). The authors suggested that hypothalamus-pituitary dysfunction could result from reversible hypophysitis or direct hypothalamic damage (Leow et al., 2005). Notably, groups of patients with CFS exhibited blunted HPA axis activity, with reduced 24-h free cortisol excretion, increased sensitivity to ACTH, and an attenuated response to CRH (Clauw and Chrousos, 1997). Therefore, patients with prolonged COVID experiencing unexplained fatigue, lethargy, malaise, orthostatic dizziness, anorexia, and apathy, particularly those with hypothyroidism features unresponsive to hydration and traditional treatments, should be evaluated for HPA axis dysfunction (Bansal et al., 2022).

Patients with long COVID experiencing general fatigue, depression, and fatigue scores were positively correlated with serum cortisol and free thyroxine (FT4) levels, respectively. Additionally, patients with general fatigue exhibited lower serum growth hormone (GH) levels and higher levels FT4 levels, while those patients with anosmia/dysgeusia had significantly lower serum cortisol levels. Higher serum thyrotropin (TSH) levels and lower FT4/TSH ratios in initially severe cases, suggested occult hypothyroidism. Furthermore, plasma adrenocorticotropin to serum cortisol ratios were decreased in patients with relatively high serum SARS-CoV-2 antibody titers. These studies together (Table 1) provide evidence that the impacts caused by the spike protein on the hypothalamic centers may be directly correlated with NeuroPASC symptoms (Sunada et al., 2022).

**Table 1:** Impacts on the hypothalamic–pituitary axis and association with NeuroPASC symptoms.

Hypothalamic–pituitary axis area	Affected function	NeuroPASC symptoms	References
Hypothalamic–pituitary–adrenal axis	Cortisol regulation and stress response	- Anxiety and emotional disturbances - Mental exhaustion and fatigue - Dysregulated stress responses	Alzahrani et al. (2021) and Ong et al. (2022)
Hypothalamic–pituitary-thyroid axis	Metabolism, energy, and cognitive functions	- Fatigue, lethargy - Memory and reasoning difficulties - Mood disorders (anxiety, irritability)	Bansal et al. (2022)
Hypothalamic–pituitary-testicular axis	Release of Gonadotropin-Releasing Hormone (GnRH)	- Sexual dysfunction (decreased libido, erectile dysfunction) - Mood changes (Irritability, Depression) - Cognitive deficits	Ardestani Zadeh and Arab (2021), Selvaraj et al. (2021), and Sauve et al. (2023)
Hypothalamic–pituitary-somatotropic axis	Secretion of growth hormone (GH)	- Chronic Fatigue Syndrome	Sunada et al. (2022)

## 4 SARS-CoV-2 spike protein and neuropathogenesis

Several studies point out that neurological symptoms or CNS damage cannot be solely attributed to viral infection (Solomon, 2021). A recent study indicates that the infection is not required for cognitive impairment in long COVID (Fernandez-Castaneda et al., 2022). These findings strongly support the participation of factors other than viral proliferation in the brain as the main cause of neurological symptoms (Muhl et al., 2020). S, S1, and N proteins are detectable in approximately 65% of patients diagnosed with PASC several months after SARS-CoV-2 infection. Of the three antigens, the S protein is detected most frequently in 60% of PASC-positive patients. S1 is detected to a lesser extent in about a fifth of patients and N is rarely detected. Antigens detection is most likely found in patients reporting PASC’s acute gastrointestinal and neuropsychiatric symptoms (Swank et al., 2023).

### 4.1 Spike and vascular dysfunction and blood–brain barrier disruption

The main underlying endothelial cell-EC dysfunction mechanisms involved in COVID-19 pathogeneses include: (i) SARS-CoV-2 infection-mediated EC apoptosis; (ii) Imbalance of the renin-angiotensin-aldosterone system (RAAS)/kallikrein-kinin system (KKS); (iii) Complement activation; (iv) Activation of inflammatory, mitochondrial oxidative stress and growth factors signaling pathways, promoting endothelial damage (Jin et al., 2020). On the other hand, EC dysfunction may result in inflammatory-immune cell infiltration and vascular leakage, leading to edema (Teuwen et al., 2020). The main proposed mechanisms can lead to: (i) the secretion of inflammatory cytokines (TNF-*α*, IL-1β, and IL-6), and chemokines, which in turn can lead to a “cytokine storm”; (ii) adhesion molecules (ICAM-1, VCAM-1, MCP-1 and VAP-1); (iii) Ang II-AT1R activation (Ferrario et al., 2005), and IL-6/ROS production (Akwii et al., 2019); (iv) KKS-B1/2R activation, increased levels of vascular endothelial growth factor (VEGF) levels and VEGFA/VEGFR2 activation (for more detail see the next topics) (Tong et al., 2020). This set of pathophysiological changes was associated with long COVID syndrome and is known as “vascular long COVID” (Zanini et al., 2024). Indeed, a relationship between the endothelial biomarkers levels and pro-inflammatory cytokine and chemokine levels was associated with the severity of pulmonary damage in COVID-19 (Osburn et al., 2022).

#### 4.1.1 Spike and brain endothelial dysfunction

Vascular long COVID can lead to prolonged neuro-inflammation, synaptic dysfunction, and disturbed neurotransmitter release, which in turn can lead to cognitive dysfunction in PASC patients (Shabani et al., 2023). Remarkably, S and S1 subunit-activated macrophages induced activation of human lung microvascular endothelial cells, increasing adhesion molecules, pro-coagulant markers, and chemokines (Rotoli et al., 2021). Moreover, the S protein has also been shown to induce VEGF production, a potent inducer of vascular permeability. This raises the possibility that vascular activation by viral infection or S protein, along with pre-existing brain diseases (which are more common in older individuals) places them at greater risk of neurological sequelae of PASC (Agrawal et al., 2022). On the other hand, the SARS-CoV-2 virus and the S protein can directly interact with the BBB, inducing pro-inflammatory effects, resulting in increased BBB permeability through damage to tight junctions (TJs) but not adherents junctions (AJs) (Suprewicz et al., 2022). Remarkably, epithelial cells expressing intracellular S protein showed to elicit an inflammatory response in macrophages when co-cultured. Furthermore, S protein can interact with Toll-like receptors, TLR2 and TLR4 receptors from macrophages, and activate signaling pathways involving PI3K, AKT, MAPK, and NF-κB, with the subsequent production of pro-inflammatory cytokines, and adhesion molecules, such as ICAM, VCAM and E-selectin (Khan et al., 2021; Zhao et al., 2021). In addition, the aforementioned cytokines and adhesion molecules can increase vascular permeability, and the severity of inflammation is closely correlated with BBB damage (Alquisiras-Burgos et al., 2021). The relevance of peripheral inflammation-induced BBB disruption is discussed by Huang et al. (2021). On the other hand, infected human brain microvascular endothelial cells (hBMECs) showed a low expression of the TJ protein and an overexpression of proinflammatory cytokines, chemokines, and adhesion molecules (Yang et al., 2022). The proinflammatory IL-1β cytokines showed VEGF expression induced by astrocytes with the subsequent metalloproteinase-9 (MMP-9)-mediated TJ protein disruption matrix (Ralay Ranaivo et al., 2011).

#### 4.1.2 Spike and glycocalyx disruption

The glycocalyx is a layer which covers luminal endothelial cells and contributes to maintain vascular homeostasis, vascular tonus, and permeability, and also modulates leukocyte adhesion and inflammation (Mockl, 2020). It was recently reported that plasma of COVID-19 patients induced glycocalyx shedding, resulting in hyperinflammation and oxidative stress (Potje et al., 2021), while the glycocalyx damage allowed S-ACE2 binding, in turn enabling viral entry (Targosz-Korecka et al., 2021). These *in vitro* findings are supported by reports about the high levels of syndecan-1 (SDC-1) in convalescent patients (Vollenberg et al., 2021), suggesting that endothelial damage persists during COVID-19 progression, and highlights an important role in long COVID and possibly of the spike protein, given its persistence in circulation after acute infection.

#### 4.1.3 Spike and pericytes dysfunction

Pericytes are contractile cells which wrap capillaries regulating tissue blood flow, in the brain, heart, and kidney. The infection of pericytes by SARS-CoV-2 underlies both the virus entry into the CNS and the appearance of neurological symptoms, due to the induction of perivascular inflammation and compromised blood–brain barrier (Robles et al., 2022). Moreover, pericytes exposed to the spike protein also can lead to vessel-mediated brain damage. Studies suggest that spike protein may reduce cerebral capillary blood flow recruiting reactive pericytes to damaged tissue, and thereby contribute to brain microvasculature injuries associated with COVID-19 (Robles et al., 2022). Indeed, spike-exposed pericytes displayed phenotypic changes associated with an elongated and contracted morphology along with an increased expression of ACE2 and contractile and myofibrogenic proteins (Khaddaj-Mallat et al., 2021a). At the functional level, S protein exposure promotes Ca^2+^ influx, the signature of contractile pericytes. Furthermore, S protein induced lipid peroxidation, oxidative and nitrosative stress in pericytes, in addition to triggering the NFk-B signaling pathway, thereby increasing the production of pro-inflammatory cytokines involved in activating and trafficking immune cells, which is also potentiated by hypoxia a condition associated to vascular disease. These findings indicate that S protein may impair the brain pericyte functions, which in turn can lead to vessel-mediated brain damage (Khaddaj-Mallat et al., 2021b). Interestingly, ACE2 treatment induced a mild pericyte-mediated capillary constriction through AT1R activation, which was enhanced by the RBD and by spike-pseudotyped viral particles in human and hamster brain tissue (Hirunpattarasilp et al., 2023).

#### 4.1.4 Mast cell activation and BBB disruption

It is suggested that neuro-PASC may (or at least partially) be caused by the activation of cerebral mast cells, which would lead to perivascular inflammation and disruption of neuronal connectivity and neuronal signal transmission (Theoharides and Kempuraj, 2023). Meningeal mast cells are capable of affecting the integrity of the BBB and promoting cerebral infiltration of T cells (Sayed et al., 2010), since mast cells are an early activator of LPS-induced neuroinflammation and BBB damage in the hippocampus (Wang et al., 2020). Furthermore, it is proposed that S protein may enter the brain directly or through mast cell activation, which then disrupts the integrity of the BBB (Theoharides and Kempuraj, 2023). In fact, spike/RBD-triggered mast cell activation was shown to induce inflammatory factors in human brain microvascular endothelial cells and microglia (Wu et al., 2024). Moreover, mast cell activation and degranulation destroyed tight junction proteins in brain microvascular endothelial cells and induced microglial activation and proliferation. Finally, full-length S protein, but not RBD, stimulated the secretion of the pro-inflammatory cytokine interleukin-1β (IL-1β), as well as the proteolytic chymase and tryptase enzymes. This effect was mediated by TLR4 for IL-1β and the ACE2 pathway for chymase and tryptase (Wu et al., 2024). These results provide strong evidence that the SARS-CoV-2/S protein contributes to inflammation by stimulating mast cells through different receptors (Tsilioni and Theoharides, 2023).

### 4.2 Spike and microglia and astrocytes activation

Microglia and astrocyte subpopulations associated with COVID-19 share features with pathological states previously observed in human neurodegenerative diseases (Yang et al., 2021). This suggests that neuroinflammatory processes during initial SARS-CoV-2 virus infection can induce reprogramming of CNS cells that contribute to neuroinflammation, neurodegeneration, and persistent neurological symptoms observed in neuro-PASC after COVID-19 acute infection (Chagas and Serfaty, 2024). Some mechanisms related to microglial deregulation are being explored: (i) the decrease in microglial levels of brain-derived neurotrophic factor (BDNF); (ii) the involvement of the NLRP3 inflammasome, linked to decreased long-term potentiation (LTP); (iii) the deregulated expression of cytokines, chemokines, and complement system molecules, interfering with adequate synaptic plasticity and inducing insufficient or excessive synaptic removal; (iv) the lack of influence of microglia on matrix remodeling which promotes E/I imbalance and compromised neural circuit maturation; and (v) the cross-talk between microglia and other circulating immune cells, such as CD4+ T lymphocytes, which further aggravates these effects. These mechanisms illustrate how abnormal plasticity contributes to the complications of neuro-PASC (Chagas and Serfaty, 2024). The microglial NLRP3 inflammasome appears as a major driver of neurodegeneration. It has been demonstrated mechanistically that S protein can activate NLRP3 in LPS-stimulated microglia in an ACE2-dependent manner. Spike protein can also prime the inflammasome in microglia through NF- κB signaling, allowing activation through ATP, nigericin, or *α*- synuclein. These findings and others (discussed below) support possible participation of microglial innate immune activation by S protein, in the increased vulnerability to the development of neurological symptoms similar to Parkinson’s disease in individuals infected with COVID-19 (Albornoz et al., 2023). Furthermore, BDNF acts on neuronal regulation, survival, and neural plasticity. Reductions in plasma and brain BDNF levels are common in patients with psychiatric and neurodegenerative diseases, possibly secondary to a state of chronic inflammation affecting the brain (Lima Giacobbo et al., 2019).

Three routes could lead to microglial activation from direct contact with viral particles: (i) through the olfactory tract; (ii) through the hematogenous and endothelial pathway, and (iii) through the blood-CSF barrier (Robichaud and Chamard-Witkowski, 2023). The S1 subunit showed to be sufficient to provoke neuroinflammation, including activation of microglia and gene expression of multiple pro-inflammatory cytokines and altered animal behavior reminiscent of neurological and cognitive symptoms in patients with COVID-19 (Frank et al., 2022). Evidence suggests that the S1 subunit can travel along nervus neurons terminals and its axons to the brain and then activate microglia by binding to TLR4, resulting in increased expression of pro-inflammatory cytokines such as IL1β and antigen-presenting molecules such as MHC-II – molecules upregulated in post-mortem brains of COVID-19 patients. Thus, a complete brain infection with viral replication is not necessary to induce neuroinflammation with neurological, cognitive, and neuropsychiatric symptoms caused by microglial activation, synapse removal, and neuronal death (Frank et al., 2022). On the other hand, systemic inflammation by hematogenous route and subsequent breakdown of the BBB allow peripheral entry of immune cells and cytokines into the CNS, activating microglia.

It is known that microglial activation and consequent astrocytic reactivity impair synaptic and myelin plasticity, inhibit hippocampal neurogenesis, promote inappropriate synaptic elimination and lead to excitotoxicity, responsible for neuronal and oligodendrocyte death (Robichaud and Chamard-Witkowski, 2023). Once overcoming the blood-CSF barrier, a spike protein’s presence in ventricles can have a delayed impact on cognitive function, recapitulating PASC. Hippocampal neuroinflammation and microgliosis have also been observed to mediate spike-induced memory dysfunction through complement-dependent synapse engagement. Genetic or pharmacological blockade of TLR4 signaling has been shown to protect animals against elimination of synapse and memory dysfunction induced by brain infusion of S protein (Fontes-Dantas et al., 2023). The astrocyte response to infection occurs by remodeling energy metabolism, which affects metabolic pathways associated with neuron nutrition and support neurotransmitter synthesis. The altered secretory phenotype of infected astrocytes impairs neuronal viability. These events can contribute to neuropathological changes, neuropsychiatric symptoms, and cognitive impairment. Although increasing evidence confirms neuropsychiatric manifestations are mainly associated with severe COVID-19 infection, long-term neuropsychiatric dysfunction has been frequently observed after mild infection (Crunfli et al., 2022). Interestingly, a Single-cell phototransfection of mRNAs encoding spike and nucleocapsid in human astrocytes resulted in RNA-dependent translation interference (Wang et al., 2024).

### 4.3 Spike-based vaccines’ effect on neuro-PASC

It has been suggested that S protein, whether by viral infection or post-vaccination, could induce pathophysiological manifestations and specifically neuroinflammation and changes in synaptic plasticity. In fact, much evidence regarding the induction of vaccination-induced adverse effects has been accumulated. The post-COVID-Vaccine-Syndrome (PACVS) has been associated with fatigue, cognitive disorders, headaches, visual alteration, and muscle pain, among other symptoms. Adverse effects on the cardiovascular and neurological systems, as well as with autoimmune and inflammatory phenomena were also reported (Finsterer, 2022; Liu et al., 2022; Rodriguez et al., 2022; Sriwastava et al., 2022). Alterations in BBB integrity have been found after COVID-19 vaccination (Cabral et al., 2022; Rastogi et al., 2022). Spike mRNA vaccine (BNT162b2 vaccine) administered during pregnancy has been shown to significantly alter WNT gene expression and BDNF levels in male and female offspring neonatal rats, suggesting that the vaccine may have an impact on key neurodevelopmental pathways (Erdogan et al., 2024). In this study, male rat exhibited autism-like behaviors characterized by a marked reduction in social interaction and repetitive behavior patterns. Furthermore, there was a substantial decrease in neuronal counts in critical regions of the rat brain, indicating potential neurodegeneration or altered neurodevelopment. Male rats also demonstrated impaired motor performance, evidenced by reduced agility and coordination (Erdogan et al., 2024). Moreover, the S1 subunit spike protein has been detected in the blood for up to 14 days, in cerebrospinal fluid (CSF) for up to 2.5 months, and in breast milk for up to 45 days in individuals after receiving one or two doses of the BTN162b2 or Moderna (mRNA-1273) vaccines (Hanna et al., 2022; Ogata et al., 2022). However, prospective studies have shown that vaccination, up to two doses, before or after SARS-CoV-2 infection reduced the risk of development of PASC-prolonged symptoms post-infection (Simon et al., 2021). Remarkably, one or two vaccination doses not only showed to reduce the risk of manifesting a more extensive range of symptoms but also the mortality (Taquet et al., 2022). Although the exact protection mechanism is unknown, a low number of doses of vaccine probably enhances the humoral (anti-S Abs) and cellular immune responses, promoting the clearance of viral reservoirs, and thereby eliminating SARS-CoV-2 antigen-mediated chronic inflammation. Nevertheless, the apparent protective effect of long COVID conferred by vaccines has recently been questioned, since high antibody titers induced by successive vaccinations showed to have opposite effects (Tsuchida et al., 2022). These latest findings raise a warning due to the occurrence of successive infection waves, along with the continuation of vaccination programs worldwide.

### 4.4 Spike and cellular brain receptors interplay-mediated neuropathogenesis

SARS-CoV-2 infects the host cell via its spike glycoprotein (Tortorici et al., 2019). After being cleaved by cellular furin protease, the S protein is incorporated into the viral surface as a homotrimer (Daly et al., 2020). Infection begins with the binding of the S1 subunit, primarily through its RBD, to the ACE2 receptor (Hoffmann et al., 2020b; Li et al., 2020b). However, SARS-CoV-2 also utilizes other surface molecules as receptors and co-receptors depending on the cell type (Gadanec et al., 2021). These include toll-like receptor 4 (TLR4), neuropilin-1 (NRP1), cluster of differentiation (CD147), glucose-regulated protein (GRP78), human dipeptidylpeptidase 4 (DPP4), RGD-binding *β* integrins, receptors of advanced glycation end products (RAGE), and transferrin receptor (TfR) (Gadanec et al., 2021). Following the initial interaction, the transmembrane serine protease 2 (TMPRSS2) and a disintegrin and metalloprotease 17 (ADAM17) mediate the cleavage of the S2 subunit, facilitating fusion of viral and cellular membranes (Hoffmann et al., 2020a).

#### 4.4.1 ACE2

ACE2 is widely expressed in the sustentacular cells of the olfactory epithelium. However, some studies have reported either the absence of ACE2 or its detection at lower levels in most olfactory receptor neurons (Bilinska et al., 2020). In contrast, high ACE2 expression has been observed in nerve terminalis, a bilateral bundle of nerve fibers extending through the subarachnoid space from the medial olfactory stria at the base of the frontal lobe to the nasal septum (Bilinska et al., 2021). An investigation into ACE2 expression of ACE2 across various human cell types and tissues, including 21 different anatomical regions of the brain, revealed that overall ACE2 expression in the brain is low compared to other tissues. The highest ACE2 expression in the brain was found in following regions: (i) the pons and medulla oblongata in the human brainstem, which contain the medullary respiratory centers (Lukiw et al., 2022); (ii) subfornical organ, area postrema, choroid plexus, and paraventricular nucleus of the thalamus and hypothalamus (Chen et al., 2020; Ong et al., 2022), with the choroid plexus showing ubiquitous ACE2 expression (Piras et al., 2022); and (iii) CVOs, especially in the paraventricular nucleus of the hypothalamus and in the choroid plexus (Ong et al., 2022).

CVOs are brain structures that regulate the body’s homeostasis through blood–brain communication (Munoz, 2022). The interaction between the S protein and ACE2 in the CVO regions has been implicated in hormonal changes and psychiatric effects, such as post-viral fatigue, sleep–wake cycle disturbances, stress, anxiety, and depression (Rosenzweig et al., 2020; Ardestani Zadeh and Arab, 2021; Jocher et al., 2022). One study found that ACE2 expression in the normal brain is more restricted to the choroid plexus and ependymal cells than other brain cell types. Interestingly, there was upregulation of ACE2 in epithelial cells in some COVID-19 patients with neurological involvement, particularly in the white matter and in patients with severe neurological symptoms (Lindskog et al., 2022). Human brain microvascular endothelial cells (hBMVEC) exhibit low levels of ACE2, but recombinant S protein increased ACE2 expression (Reynolds and Mahajan, 2021). Similarly, S protein stimulation led to increased ACE2 expression in human brain vascular pericytes (Khaddaj-Mallat et al., 2021a). Although studies using single-cell RNA sequencing (scRNA-seq) detected low ACE2 levels in brain cells, relatively high ACE2 expression was found in some components of the neurovascular unit, particularly in cerebral pericytes (Muhl et al., 2020). Additionally, prominent capillaries and tanycytes of the hypothalamus express TMPRSS-2 and ACE2, which may facilitate SARS-CoV-2 infection in brain tissue (Ternier et al., 2020). These findings suggest that ACE2 expression in vascular mural cells, such as pericytes, makes them a more likely target of SARS-CoV-2 infection.

#### 4.4.2 S/ACE2-*β* integrin interplay in BBB endothelial cells

The ACE2 protein functions both as the primary receptor for SARS-CoV-2 and as a modulator of the RAAS system. ACE2 converts angiotensin (Ang) I into Ang 1–9 and Angiotensin II (Ang II) into Ang 1–7, leading to reduced Ang II levels and attenuated activation of the AT1R receptor (Donoghue et al., 2000). Upon viral infection, ACE2 internalization disrupts RAAS balance, resulting in increased Ang II levels and enhanced AT1R activation. The Ang II/AT1R axis mediates various biological responses, including vasoconstriction, the release of chemokines and pro-inflammatory cytokines (MCP-1, IL-1β, IL-6, TNF-*α*, and IFN-*γ*), inhibition of anti-inflammatory cytokines like IL-10, and increased ROS production (de Queiroz et al., 2020). The elevation of chemokines can lead to the activation, chemoattraction, and infiltration of T lymphocytes (LT), neutrophils, and monocytes into brain tissue. Additionally, activation of Ang-II/AT1R axis can promote ADAM17 activation, RAGE1 transactivation and NF-κB-mediated inflammatory responses in various cell types (Pickering et al., 2019). ADAM17 activation results in cleaving and shedding the ACE2 ectodomain, further reducing cell surface ACE2 levels, increasing Ang II/AT1R axis activity, and creating a positive feedback loop. Overactivation of ADAM17 also leads to shedding inflammatory factors such as TNF-*α*, IL-6R, IL-6 and TNFR1/2, and VEGF, which may contribute to acute inflammatory response and the activation of the coagulation cascade (Zipeto et al., 2020).

**Figure 2:**
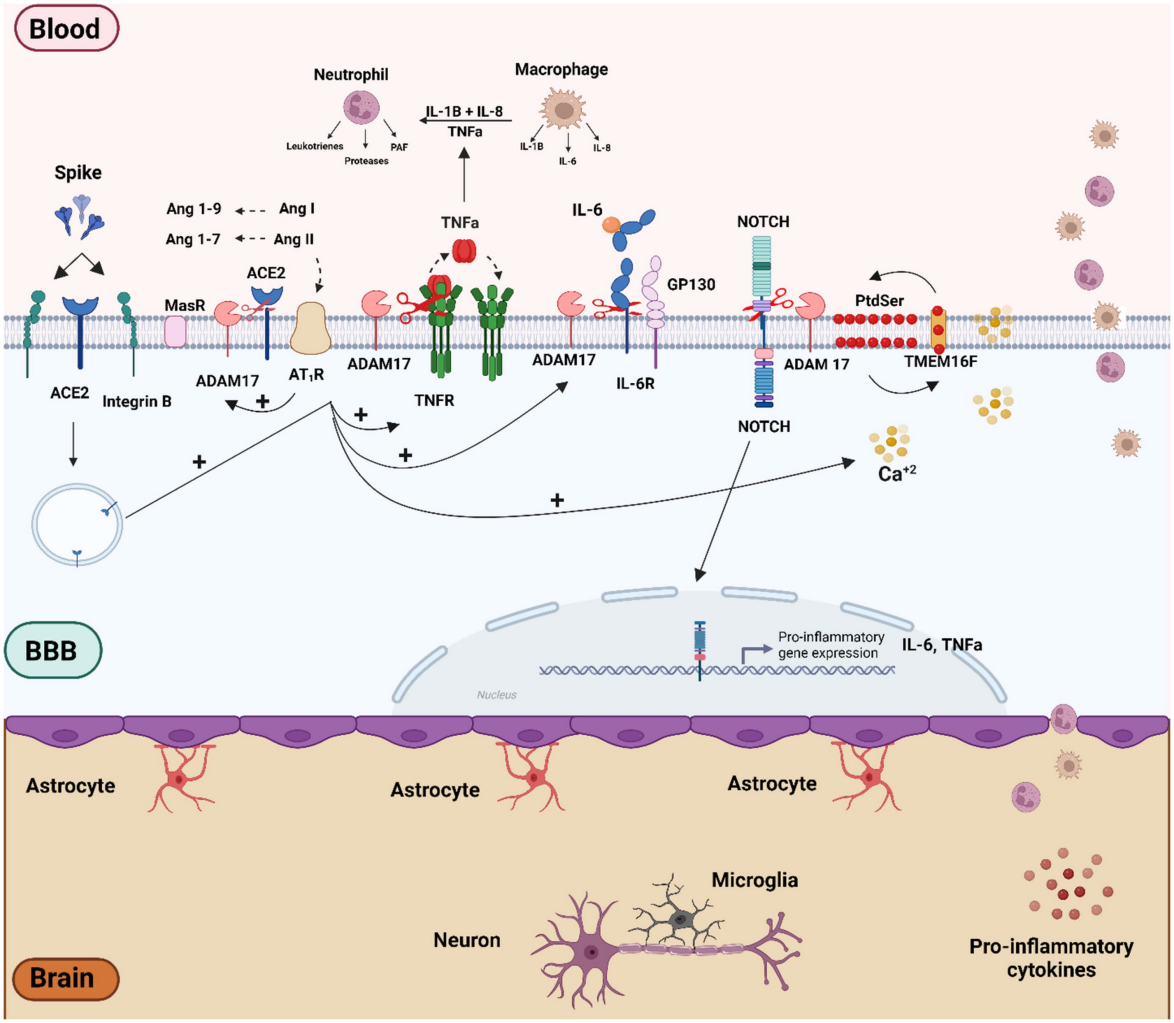
Schematic overview of the molecular mechanisms related to SARS-CoV-2 and S protein effect on vascular dysfunction and BBB disruption. Vascular cells and pericytes of the BBB can be activated by SARS-CoV-2 infection or by the binding of the spike protein to ACE2 and b-integrin receptors. The imbalance of the RAS pathway mediated by ACE2 downmodulation can lead to activation of the Ang II/AT1R axis. Both AT1R activation and viral infection itself can trigger several intracellular signaling, such as Ca^2+^ influx and activation of TMEM16F-scramblase and externalization of PtdSer to the outer cell membrane, culminating in the activation of ADAM17. The ADAM17 sheddase activity, together with AT1R-mediated RAGE activation may play a preponderant role in the inflammatory process by activating inflammatory factors such as NOTCH and NF-kB proteins, which in turn can lead to the production of pro-inflammatory factors such as TNF-*α*, IL-6, R-IL6, IL1-*β*. Figure created with BioRender.com.

Recent research has shown that ADAM17-dependent regulation of ACE2 expression is linked to the expression of the bradykinin B1 receptor in primary cultures of hypothalamic neurons (Parekh and Sriramula, 2020). Furthermore, ADAM17 activation can inhibit axon remyelination by blocking neuregulin 1 type III (NRG1-III). Axon demyelination may result from increased ROS and cytokines, and potentially from direct viral damage to both the CNS and peripheral nervous system (PNS) (La Marca et al., 2011). The association between elevated ADAM17 expression and activity with poorer clinical outcomes and increased mortality underscores the significance of this metalloproteinase in COVID-19 pathophysiology (Sun et al., 2024). The interaction between the spike (S) protein and ACE2 can also activate intracellular mechanisms that involve ADAM17. Specifically, the S1 subunit binding with ACE2 has been shown to induce ADAM17-mediated shedding of ACE2 and IL-6 shedding in adipocytes (Ardiana et al., 2023). Furthermore, the interaction of the S protein’s receptor-binding domain (RDB) with ACE2 or β-integrin can trigger sequential intracellular signaling events. An increase in cytoplasmic Ca^2+^ initially activates TMEM16/Anoctamin-6 “scramblases,” leading to the translocation of phosphatidylserine (PtdSer) to the outer membrane leaflet (Braga et al., 2021). The translocation of PtdSer can then activate ADAM17 on the cell membrane and the extrinsic coagulation pathway through tissue factor (TF) (Sommer et al., 2016). Notably, transient increases in cytoplasmic Ca^2+^, TMEM16 activity, and PdtSer translocation have also been observed in brain pericytes, stimulated with both the full-length S protein and the RBD fragment (Khaddaj-Mallat et al., 2021a). Finally, ADAM10/17 is also involved in NOTCH activation, an acute phase inflammatory protein that can induce proinflammatory cytokines production such as TNF-α and IL-6 (Bozkulak and Weinmaster, 2009). Thus, spike protein-mediated ADAM17 activation in cerebral vascular endothelium emerges as a critical factor in pathogenesis, potentially leading to inflammation, vascular dysfunction, leakage, edema, and activation of the coagulation cascade (Figure 2).

#### 4.4.3 S/TLR4 interplay in microglia cells

Toll-like receptors (TLRs) are essential for initiating the innate immune response to infection, stress or injury, and are activated by various pathogen-associated molecular patterns (PAMPs) (Kawasaki and Kawai, 2014). Research has demonstrated that SARS-CoV-2 S protein binds to TLR4 with greater affinity than to ACE2 (Choudhury and Mukherjee, 2020), leading to aberrant signaling that contributes to the hyperinflammatory response seen in COVID-19 patients (Aboudounya and Heads, 2021). *In vitro* studies have also shown that the S protein activates TLR2/4 in phagocytic cells, stimulating production of pro-inflammatory mediators (Khan et al., 2021). Microglia, the most abundant immune cells types in the CNS, play a pivotal role in neuroinflammatory diseases (Dheen et al., 2007). Microglial cells can exhibit both protective and harmful activities during viral encephalitis, depending on the infection stage (Chen et al., 2019). Injection of the S1 subunit into the brain via intra-cisterna magna (ICM) modulated the neuroimmune gene expression across several brain regions (including hypothalamus, hippocampus, and frontal cortex) affecting genes such as *Iba1, Cd11b, MhcIIα, Cd200r1, Gfap, Tlr2, Tlr4, Nlrp3, Il1b, and Hmgb1,* as well as protein levels (IFN-*γ*, IL-1β, TNF, CXCL1, IL-2, IL-10) for up to 7 days post-S1 treatment (Frank et al., 2022). Additionally, the S1 subunit induced similar behavioral changes to those observed in PASC. The infusion of the S protein into the brains of mice also impaired cognitive function through complement-mediated synapse destruction, mimicking post-COVID-19 syndrome (Fontes-Dantas et al., 2023).

**Figure 3:**
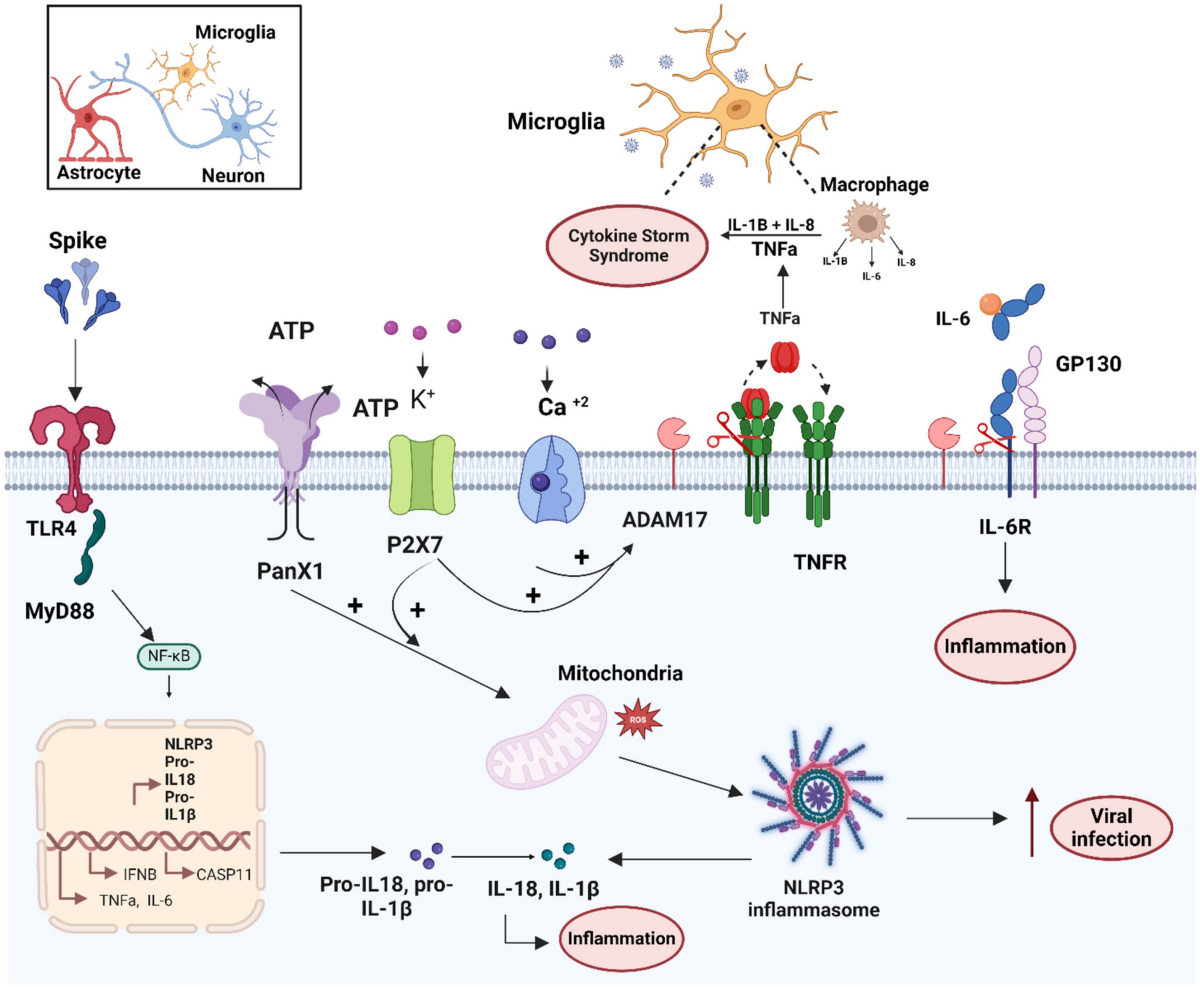
Schematic overview of the molecular mechanisms related to microglia activation by the SARS-CoV-2 and S protein. Microglial cells can mainly be activated by SARS-CoV-2/S spike protein through the TLR4-signaling pathway. In addition, the S protein enhances activity of the ATP+ ion channel, and PANX1 and purinergic receptor P2X7/K+, which together with calcium influx can lead to ADAM17 activation and mitochondrial dysfunction. Both TLR4 and PANX1/P2X7 activation can lead to activation of NF-Kb transcription factor and inflammasome system activation. These mechanisms together lead to the production of pro-inflammatory cytokines such as TNF-α, IL-6, RIL-6, IL1-β and IL-18, involved in the cytokine storm and the increase of viral infection. Figure created with BioRender.com.

TLR4 signaling has been identified as the primary pathway responsible for long-term cognitive dysfunction after COVID-19 infection in humans, with the GG genotype TLR4-2604G > A (rs10759931) serving as a marker for poor cognitive outcome (Fontes-Dantas et al., 2023). *In vitro* studies further demonstrated that the S1 subunit induces pro-inflammatory gene expression (TNF-α, IL-6, IL-1β and iNOS/NO) and NF-kB activation in mouse BV-2 microglia cell lines (Olajide et al., 2022). S1 also increased the expression and activation of purinergic receptors, such as P2x7, in BV-2 cells, and activated the TLR4 signaling pathway (Olajide et al., 2022). Notably, Pannexin-1/ATP-dependent P2X7 activation acts as a second activation signal of the inflammasome system following TLR4-NFkB pathway activation (Yue et al., 2023). The P2x7 and NLRP3 inflammasome pathways are essential for SARS-CoV-2 infection and involved in the COVID-19 pathogenesis (Lecuyer et al., 2023). On the other hand, activation of PANX-1/ATP-dependent P2 purinergic receptors can also induce ADAM17/10 activation via the ERK and PI3K signaling pathway, along with PtdSer translocation through increased intracellular Ca^2+^ influx (Pupovac et al., 2015). Thus, S-TLR4/Pannexin1/ATP/P2X7 and S-ACE2/Ca^2+/^TMEM16/PtdSer pathways may synergistically enhance ADAM17 activity (Sommer et al., 2016). Consequently, ADAM17 activation mediated by the TLR4 and ACE2 receptor signaling may represent another mechanism by which SARS-CoV-2 induces the neuroinflammation and micro-coagulopathies observed in neuro-PASC (Figure 3; Patra et al., 2020).

#### 4.4.4 S/NRP1-CD147-DPP4 interplay in astrocytes

NRP1 is expressed in the olfactory epithelium and in certain brain tissues and cells, highlighting its potential role in CNS infection and neuro-pathogenesis (Cantuti-Castelvetri et al., 2020; Davies et al., 2020; Lechien et al., 2020; Hopkins et al., 2021; Kong et al., 2022). While astrocytes do not exhibit detectable ACE2 levels, they notably do express elevated NRP1 levels (Potokar et al., 2023). Quantitative RNA and protein data from pericytes and astrocytes extracted from the BBB indicate moderate ACE2 and TMPRSS-2 expression levels in astrocytes, but high NRP1 expression levels, with pericytes showing variable protein expression of ACE2, NRP1, and higher TMPRSS-2 mRNA expression (Malik et al., 2023). Although both astrocytes and pericytes permit viral infection, evidence suggests that astrocytes produce higher viral levels in culture supernatants than pericytes.

The importance of NRP1-mediated SARS-CoV-2 infection in astrocytes has been demonstrated by histopathological and molecular studies using an anti-NRP1 neutralizing antibody and siNRP1 (Crunfli et al., 2022). Moreover, SARS-CoV-2 infection of astrocytes leads to increased expression of IFN and inflammatory mediators, along with decreased expression of ions and neurotransmitter transporters. These events result in the dysfunction and death of uninfected neurons, contributing to CNS deficits (Kong et al., 2022). Interestingly, both the expression levels and the percentage of astrocytes expressing NRP1 mRNA were higher in astrocytes from COVID-19 patients compared to controls (Crunfli et al., 2022).

**Figure 4:**
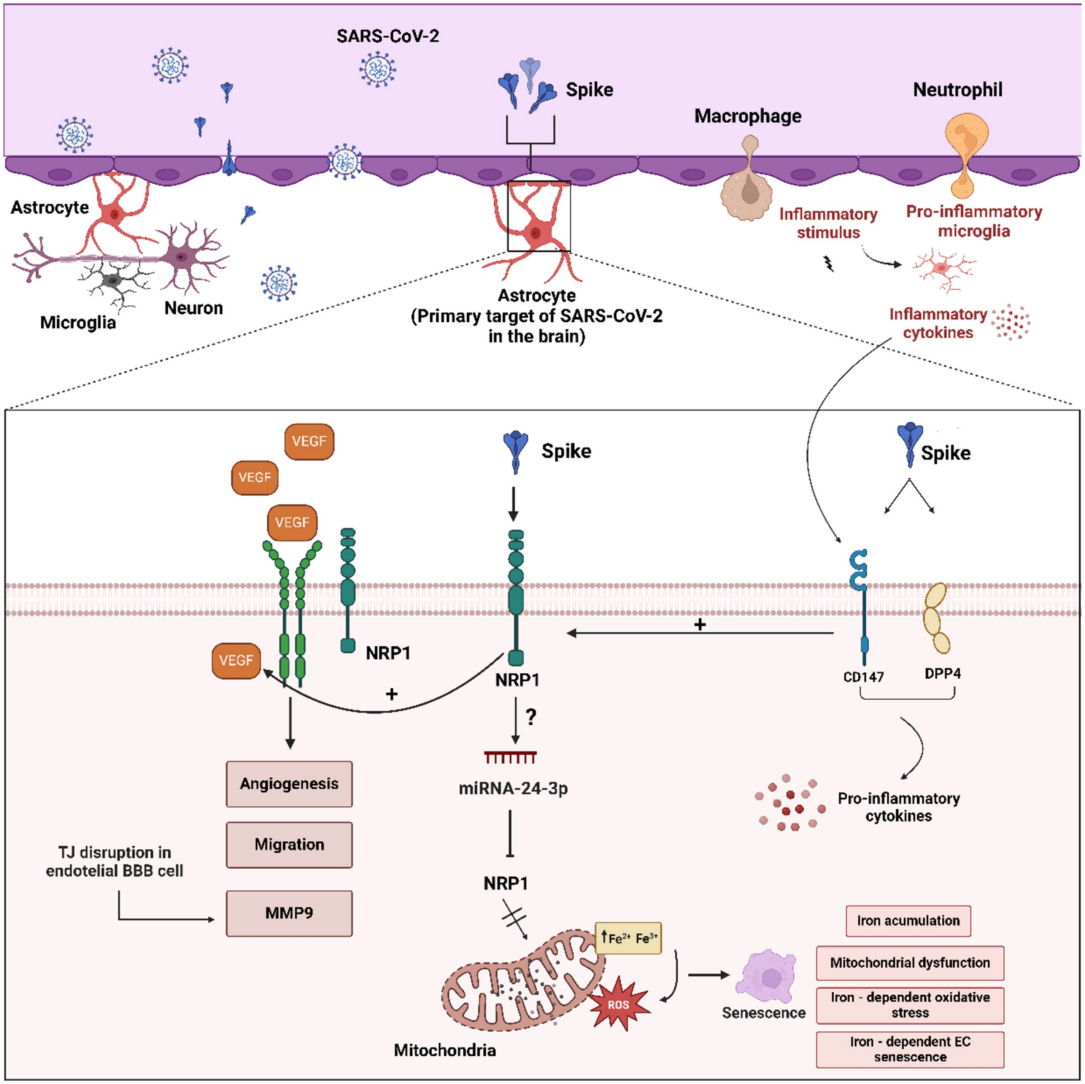
Schematic overview of the molecular mechanisms related to astrocytes activation by the SARS-CoV-2 and S protein. “Astrogliosis” astrocyte activation can be mediated by interplay between CD147/DPP4 and NRP1 receptors and SARS-CoV-2 and S protein. CD147/DPP4 activation may enhance proinflammatory cytokines, VEGF production and NRP1 activation. Furthermore, VEGF-VEGF-R2/NRP1 is able to induce angiogenesis, migration and disruption of TJ proteins in BBB capillary endothelial cells. In addition, excess VEGF can activate the ADAM9/10 metalloproteinases with the consequent release of the extracellular domain of NRP1 and cytoplasmic tail, inhibiting the VEGF-NRP1 signaling pathway in a negative feedback fashion. Figure created with BioRender.com.

NRP1 binds to vascular endothelial growth factor A (VEGF-A) and alternatively to viruses such as Epstein–Barr virus (EBV) and human T-cell lymphotropic virus-1 (HTLV-1) (Wang et al., 2015; Mercurio, 2019). Thus, NRP1 plays a critical role in vascular angiogenesis, promoting growth, survival, and self-renewal, and is also involved in axonal guidance in both the CNS and peripheral nervous system through interactions with VEGFR2 in vascular endothelial cells (Raimondi and Ruhrberg, 2013; Mercurio, 2019). Interestingly, VEGF upregulates the ADAM9/10-mediated NRP1 cleavage, releasing both extracellular and cytoplasmic fragments. The cytoplasmic fragment inhibits angiogenesis and migration induced by VEGF/VEGFR-2/NRP1 axis through a negative feedback mechanism (Figure 4; Mehta et al., 2018). This phenomenon may be significant in COVID-19 pathogenesis, as elevated VEGFA levels have been observed in the lungs of deceased COVID-19 patients (Ackermann et al., 2020). Conversely, downregulation of NRP1 has been associated with iron accumulation, inhibition of cellular growth, and immunosenescence, suggesting a possible role for ADAM9/10-mediated NRP1 shedding in PASC symptoms (Issitt et al., 2019; Hanson et al., 2024).

In addition to NRP1, the S protein also binds with high affinity to CD147 and DPP4/CD26 (Li et al., 2020a). Expression of DPP4 and CD147 increases in response to inflammation in reactive astrocytes, potentially significantly contributing to viral binding and entry into astrocytes (Andrews et al., 2022). The induction of inflammatory cytokines may in turn lead to increased VGEFA levels. Therefore, upregulation of viral receptors in astrocytes may act as a positive feedback mechanism during viral infection (Figure 4; Potokar et al., 2023).

### 4.5 Spike-cellular receptors interplay hypothesis on neuro-PASC pathophysiology

This section introduced comprehensive data that supports the role of the SARS-CoV-2/S protein in the pathophysiology of neuro-PASC. Indeed, the interaction of the spike protein with specific cellular receptors in various CNS cell types - such as endothelial cells, mast cells, pericytes, microglia, and astrocytes - may be critical in the development of the pathophysiological mechanisms associated with neuro-PASC symptoms. The proposed molecular mechanisms resulting from interactions between the spike protein and specific cellular receptors in various CNS cells and tissues can be summarized as follows: (i) The interplay between the spike protein and ACE2/*β* integrin in BBB endothelial cells may lead to ACE2 downregulation, increased AngII/AT1R activity, activation of ADAM17/NOTCH, and production of pro-inflammatory cytokines; (ii) The interaction of the spike protein with TLR4 in microglial cells can activate NFk-B/Panx1/P2x7, leading to subsequent NLRP3/ADAM17 activation and the production of pro-inflammatory cytokines; (iii) The S/NRP1-CD147-DPP4 interaction in astrocytes involves NRP1/VGEF modulation, which causes mitochondrial dysfunction and cellular senescence, while microglial pro-inflammatory cytokines can induce upregulation of CD147 and DPP4, resulting in increased expression of NRP1.

## 5 Concluding remarks and future directions

Despite the important advances in preventing severe COVID-19 cases through vaccination, the emergence of long COVID syndrome, and especially neuro-PASC, represents a new challenge. Thus, herein we have provided comprehensive evidence to better understand the physiological processes behind neuro-PASC which highlights the activation of astrocytes and pericytes in vascular dysfunction. This dysfunction may lead to the disruption of the BBB, as well as BBB-deficient regions crucial for body homeostasis based on communication between blood and brain. Therefore, neuro-PASC appears to manifest as a disorder of the blood–brain interface.

In this scenario, the SARS-CoV-2 spike protein emerges as a significant contributor to dysfunction in endothelial cells, glycocalyx, pericytes, and astrocytes. The spike protein can also interact with secretory and sensory nuclei, such as those in the hypothalamus, influencing hormonal regulation and the central nervous system’s equilibrium. Moreover, the molecular mechanisms underlying neuro-PASC effects may be due (or at least in part) to the induction of inflammatory processes and micro-coagulopathies triggered by S and/or S1 proteins binding to viral receptors expressed at the brain endothelial and tissue levels. In summary, the comprehensive data presented here highlights the need to further investigate the molecular mechanisms underlying Neuro-PASC, mainly related to S protein, and especially given the ongoing infection waves and global vaccination efforts centered around the S protein.

## Data Availability

The original contributions presented in the study are included in the article/supplementary material, further inquiries can be directed to the corresponding author.
